# Elevated MRE11 expression associated with progression and poor outcome in prostate cancer

**DOI:** 10.7150/jca.31454

**Published:** 2019-07-10

**Authors:** Jun Wang, Wen-Hao Xu, Yu Wei, Yao Zhu, Xiao-Jian Qin, Hai-Liang Zhang, Ding-Wei Ye

**Affiliations:** 1 Department of Urology, Fudan University Shanghai Cancer Center, Shanghai 200032; 2 Department of Oncology, Shanghai Medical College, Fudan University, Shanghai 20032, P.R. China

**Keywords:** MRE11, DNA repair, homologous recombination, prognosis, prostate cancer

## Abstract

**Objective**: Growing evidence has proved that MRE11, a protein underpinned to be involved in DNA double-strand break (DSB) repair process, is correlated with cancer outcomes. However, its role in prostate cancer (PCa) remains unclear. This study aimed to investigate the expression of MRE11 in tumor tissue and defining its value in predicting prognosis of PCa patients.

**Methods**: A total of 578 patients from two cohorts were enrolled in this study. Distribution of categorical clinical-pathological data together with levels of MRE11 expression was compared with χ^2^-test in a contingency table. Immunohistochemical (IHC) staining and evaluation was detected from 78 paired PCa and adjacent normal tissues. Partial likelihood test from univariate and multivariate Cox regression analysis was developed to address the influence of independent factors on disease-free survival (DFS) and overall survival (OS) in two cohorts. The Kaplan-Meier method and log-rank test were performed to assess the survival benefits between discrete levels. Set Enrichment Analysis (GSEA) was performed to select related genes and pathways from The Cancer Genome Atlas (TCGA) database.

**Results**: In the current study, we demonstrated that MRE11 was highly expressed in PCa compared with normal tissues (*P=*0.011). In addition, in the TCGA cohort, the median DFS in patients with IHC positive and negative MRE11 expression levels was 24.5 and 30.6 months, and median OS was 28.7 and 33.0 months, respectively. In FUSCC cohort, median DFS in patients with IHC positive and negative MRE11 expression was 28.0 and 35.6 months. Furthermore, survival curves suggested that PCa patients with elevated MRE11 expression levels showed poorer OS (*P*=0.019) in TCGA cohort and poor DFS (*P*=0.047) in FUSCC cohort.

**Conclusion**: In conclusion, our study reveals that elevated MRE11 expression is significantly correlated with cancer progression and poor survival in PCa patients. These data suggest that MRE11 may act as an oncoprotein and a promising prognostic marker for PCa patients.

## Introduction

Prostate cancer (PCa) has become the most frequently diagnosed cancer and the fifth leading cause of cancer death in men, accounting for approximately 13.5% incidence and 6.7% mortality of all cancer deaths 1. It is estimated to grow to 1.3 million new cases of PCa in 2018 worldwide, especially in countries with higher socioeconomic development 1-3. Hence, understanding of the key genes and underlying molecular mechanisms that regulate proliferation and invasion of PCa is significant to promote early diagnosis, develop individual therapies and predict prognosis 4, 5. However, only limited information of the available researches can be applied to the explanation of aggressive progression in PCa patients 5. Therefore, new prognostic biomarkers and molecular alterations involved in the initiation and progression of PCa must be urgently identified to realize individualized precise therapeutic regimen.

Meiotic recombination 11 (MRE11), a human ortholog of MRE11A that encodes a nuclear protein, is supposed to be involved in the repair of DNA double-strand break (DSB) 6. In this organism, MRE11 protein interacts with the Rad50 recombinase and consists of MRE11-RAD50-NBS1 (MRN) complex, which results in active recruitment of DNA damage repair pathways exposed to ionizing radiation 7, 8. Synergistically with the function of ATM 9, this complex phosphorylates protein H2AX, p53, CHK2 to accelerate DSB repair and active cycle checkpoint 10, 11. Meanwhile, during DSB repair, MRE11 participates in the amplification of apoptosis signaling if irreversible damage appears. To avoid incomplete repair of DNA damage, body cells developed homologous recombination to ensure the integrity of the genome 12. Similarly, programmed DSB is required to initiate homologous recombination during meiosis process, in which MRE11 served an essential role for DSB repair 13.

DSB repair is an indispensable process to support genomic integrity, and significant genes are activated when DNA damage occurs 14-16. In most cases, deficient expression of DNA repair genes and inevitable replication error increase the quantity of un-repaired DSB, leading to mutant and further tumorigenesis 17. Interestingly, Sharma et al. found MRE11A participated in microhomology-mediated alternative end joining (MMEJ), one of the major but error prone DSB repair pathways in higher eukaryotes 18. Therefore, these observations indicated MRE11 over-mediated DSB repair may lead to imprecise homologous recombination and elevated risk of carcinoma. For example, Yuan et al. suggested that MRE11A was a novel oncoprotein and was significantly associated with malignant behavior trend in breast cancer 19. However, the prognostic value of MRE11 in PCa was merely documented.

To investigate the MRE11 expression in tumor tissue and defining its value in PCa patients, we enrolled 78 patients who have received previous radical prostatectomy in our institution and validate its role in 500 patients in the Cancer Genome Atlas (TCGA) database. We hypothesized that the oncogenic activity of MRE11 correlated with poor prognosis and might be a potential therapeutic target of PCa.

## Materials and Methods

### Patients and Variables

The inclusion criteria were as follows: patients were pathologically diagnosed with prostatic adenocarcinoma; patients had not received chemical treatment or physical therapy before surgery. Patients with an age < 18 or a life expectancy < 1 year were excluded in this study. Finally, a total of 78 PCa patients, who have underwent previous radical prostatectomy in the Department of Urology of Fudan University Shanghai Cancer Center (FUSCC) (Shanghai, China) from October 2010 to December 2012, and 500 PCa patients from TCGA database were consecutively recruited in analyses, with electronic medical records or pathology reports available. Clinical and pathological parameters, specifically age at surgery, TNM stage, AJCC stage and Gleason score, in two cohorts were summarized in Table 1. Tissue samples, including PCa and normal tissues, were collected during surgery and available from FUSCC tissue bank. All of the study designs and test procedures were performed in accordance with the Helsinki Declaration II. The Ethics approval and consent to participate of the current study were approved and consented by the ethics committee of FUSCC.

### Immunohistochemical (IHC) staining and evaluation

Immunostaining of MRE11 was performed using a mouse monoclonal anti-MRE11 antibody (1:3000, Cat. ab214, Abcam, USA). Positive or negative staining of a certain protein in one FFPE slide was independently assessed by two experienced pathologists, and determined as follows. The staining intensity level was graded from 0 to 3. Samples with no staining, weak, median and strong staining denote to the level of 0, 1, 2 and 3. Based on the coverage percentage of immunoreactive tumor cells (0%, 1-25%, 26-50%, 51-75%, 76-100%), the staining extent was ranging from 0 to 4. The overall IHC score grading from 0 to 12 was evaluated according to the multiply of the staining intensity and extent score. Negative staining represented grade 0 to 3 and positive staining from 4 to 12 for each sample. All examples were classified in three groups (Gleason<7, Gleason=7 and Gleason>7) to confirm differential expression of MRE11.

### Statistical analysis

To figure out the associations of different MRE11 mRNA expression sets with clinicopathological characteristics, χ^2^-test was performed to compare the distribution of categorical data between groups. Scattered plot was utilized to present the differential expression of MRE11 in normal or prostate tissues. The primary end point was overall survival (OS) representing a certain period of time, which was evaluated from the date of radical prostatectomy to the date of death or last follow-up in PCa patients. Disease-free survival (DFS), as the secondary end point, was the length of time from the initiation of curative treatment when no disease can be detected until the date of progression or the start date of a second-line treatment or the date of death, whichever occurred first. The follow-up duration was estimated using the Kaplan-Meier method with 95% confidence interval (CI) and log-rank test in separate curves. Univariate and multivariate analysis were performed with Cox logistic regression models to find independent predictors. Cox regressions on 500 participants enrolled in TCGA cohort were independently analyzed to evaluate confounding covariates including age, TNM stage, Gleason score and MRE11 expression on survival. In FUSCC cohort, AJCC stage was analyzed in evaluation as supplements. Statistics analyses were performed with SPSS software (version 23.0, Inc, Chicago, IL, USA). All hypothetical tests were two-sided and *P*-values less than 0.05 were considered significant in all tests.

Datasets from the Cancer Genome Atlas (TCGA) database were implemented with GSEA method using the Category version 2.10.1 package. For each separate analysis, Student's-t-test statistical score was performed in consistent pathways and the mean of the differential expression genes was calculated. A permutation test with 1000 times was used to identify the significantly changed pathways. The adjusted *P* values (adj. *P*) using Benjamini and Hochberg (BH) false discovery rate (FDR) method by default were applied to correct for the occurrence of false positive results. Significant related genes were defined with an adj. *P* less than 0.01 and FDR less than 0.25 20.

## Results

In this study, research was conducted in two series. In the first series, differential expression of MRE11 in normal and prostate tissues was analyzed; in the second series, progression and prognostic value of MRE11 expression in PCa patients were assessed.

### Clinicopathological characteristics of the cohorts

As shown in Table 1, patients with increased MRE11 expression significantly correlated with elder patients (*P*=0.021) in FUSCC cohort and higher Gleason score (*P*=0.008) in TCGA cohort. Chi-square test showed that baseline data were balanced on the distribution of categorical data, including TNM stage and AJCC stage (*P*>0.05).

### Expression of MRE11 in FUSCC

To analyze the MRE11 expression profile of PCa tissue, IHC revealed staining distribution concerning different Gleason score between normal and tumor tissues. The scatter plot of IHC score revealed that elevated MRE11 was significantly expressed in prostate tumor tissues (*P*=0.011).

### Cox regression analyses and survival outcomes of two cohorts

In the multivariate models of FUSCC and TCGA cohort in Table 2, traditional prognostic factors, specifically pathological T stage, was still relevant to DFS in PCa patients, indicating a fine representativeness of the population in the cohort of current research. In FUSCC cohort, high Gleason score was significant correlated with poor DFS (*P*<0.001) in multivariate model of Cox regression analyses. Importantly, subgroups of MRE11 expression (IHC negative vs. IHC positive) showed that MRE11 amplification significantly associated with poor DFS (*P*=0.048) for PCa patients in FUSCC cohort (Table 2). As shown in Table 3, univariate and multivariate analysis of OS in TCGA cohort indicated that Gleason score was significantly associated with OS (*P*=0.019) merely in univariate Cox regression. Increased MRE11 expression profiles was suggested to have prognostic value in both univariate (*P*=0.047) and multivariate (*P*=0.046) analysis. The other factors, including age and TNM stage, were not assessed as prognostic indicators of OS in our study (*P*>0.05) (Table 2 and Table 3).

Survival curves suggested that PCa patients with elevated MRE11 expression levels showed poorer OS (*P*=0.019) in TCGA cohort and poor DFS (*P*=0.047) in FUSCC cohort. In addition, in TCGA cohort, median DFS was 26.9 months and OS was 30.5 months respectively. The median DFS in patients with IHC positive and negative MRE11 expression levels were 24.5 and 30.6 months, and median OS was 28.7 and 33.0 months, respectively. In FUSCC cohort, data on OS was not available due to the favorable prognosis of PCa patients, while median DFS was 30.7 months. Median DFS in patients with IHC positive and negative MRE11 expression was 28.0 and 35.6 months.

**Figure 3:**
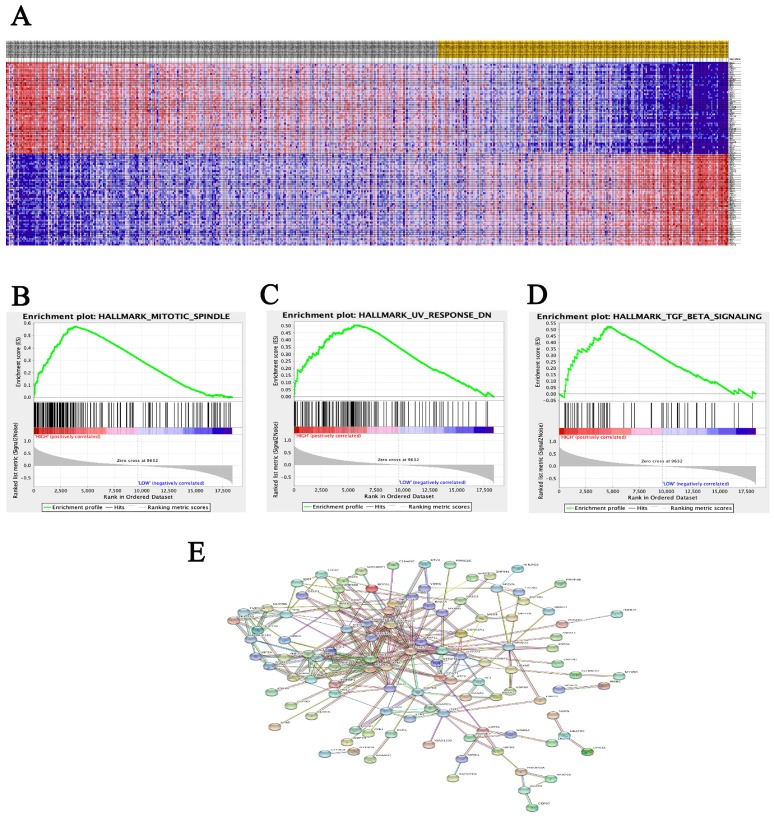
Datasets from TCGA database were implemented with GSEA method. For each separate analysis, Student's-t-test statistical score was performed in consistent pathways and the mean of the differential expression genes was calculated. A permutation test with 1000 times was used to identify the significantly changed pathways. The adjusted P values (adj. P) using Benjamini and Hochberg (BH) false discovery rate (FDR) method by default were applied to correct for the occurrence of false positive results. The significant related genes were defined with an adj. P less than 0.01 and a FDR less than 0.25.

A total of 100 significant genes were obtained from GSEA, and the genes with positive correlation were plotted. Besides, MRE11 was found involved in the most significant pathways including mitotic spindle, UV response and transforming growth factor beta (TGF-β) signaling pathways. The details were illustrated in Figure 3.

## Discussion

In our present study, we performed IHC and detected survival benefits to investigate whether MER11 has potential prognostic value in PCa. We observed MRE11 expression markedly enhanced in prostate cells was associated with malignant behavior. Collectively, our data demonstrated that high level of MRE11 expression was associated with high risk of recurrence rate and decrease patient survival. It opens up a novel way for MRE11 expression to affect the pathogenesis of PCa by underlying DNA damage variation.

In human malignancy, the MRN (MRE11-RAD50-NBS1) protein complex plays a vital role in DSB repair foci and cell cycle control, which makes it responsible for the genome stability 8. Relatively, heterozygous mutation only contributes a limited fraction of tumorigenesis, and aberrant molecular variation of MRN is more frequent and of great clinical significance 21, 22. Previous studies had reported the role of MRE11 in cell survival and proliferation 23, and malignant cancer behavior was significantly correlated with elevated MRE11 expression phenotypes in breast 19, 24, 25, gastric 26 and rectal cancer 27, 28. Similarly, it was noteworthy that increased recurrence rate and chemoresistance of certain cancers correlated with high expression level of MRN complex 28-30. Moreover, deleterious mutation-induced over-expressed RAD50 and NBS1 were associated with undesirable survival benefits in PCa patients, while independent prognostic value of MRE11 was rarely documented 31, 32.

In this study, data analysis form two cohorts support our hypothesis and clearly detect high expression of MRE11 in tumor tissues for PCa patients, leading to increased disease recurrence rates and decreased overall survival. Meanwhile, GSEA analysis illustrated that MRE11 involved in the most significant pathways including mitotic spindle, UV response and transforming growth factor beta (TGF-β) signaling pathways were enriched in PCa samples. Besides participating in DSB repair pathways, MRE11 also interacts with MMAP, expressed by mitosis-specific MRN complex, to maintain optimal genome stability during mitosis 33. In addition, the ionizing-radiation induced DSB activates a complex co-network of proteins. MRN complex results in active recruitment of DNA damage repair pathways 7, 8. Recruitment of repair factors is a prerequisite for initiation of DNA damage repair by the homologous recombination pathway. It was reported that Smad7 and SPTBN1 have independent effect on driving DNA repair process, and regulates TGF-β signaling as well 34, 35.

Strength of our study lies in our first attempt to investigate the role of MRE11 as a prognostic factor of PCa. Relationship between MRE11 and PCa was rarely documented, while it is noteworthy that MRE11 is a confirmed repair factor in DSB response process and estimated highly expressed in many cancer patients 11. With this in mind, in the FUSCC cohort of our study, we found dramatic MRE11 IHC score contrast between 78 paired tumor and adjacent normal tissues, and first demonstrated that PCa patients with elevated MRE11 expression had shorter DFS, which was similar to the validation in the TCGA database with 500 PCa patients. On the other hand, to further explain the underlying ability of invasion and metastasis of MRE11, data from public database were implemented with GSEA analysis to identify significant genes and pathways, which might clarify the correlation triggering carcinogenesis.

At the same time, there are some limitations proposed in this study as follows. Firstly, retrospective nature of the data set is inevitable, including relatively small sample size, poor population variety, and selected bias from FUSCC cohort. However, realizing this limitation of our demographic group, we collected data from TCGA to provide more plausible evidence for our conclusion. Secondly, our research failed to deeply clarify the underlying mechanism of MRE11 involved in PCa. Thirdly, data from FUSCC cohort miss the overall survival values until the last follow-up.

## Conclusion

In conclusion, our study first reveal that elevated MRE11 expression is significantly correlated with cancer progression and poor survival in PCa patients. These data suggest that MRE11 may act as an oncoprotein and a promising prognostic marker for PCa patients. In this regard, more validation cohorts and further elucidation are required to identify all value of MRE11 and its clinical application for PCa patients.

## Figures and Tables

**Figure 1:**
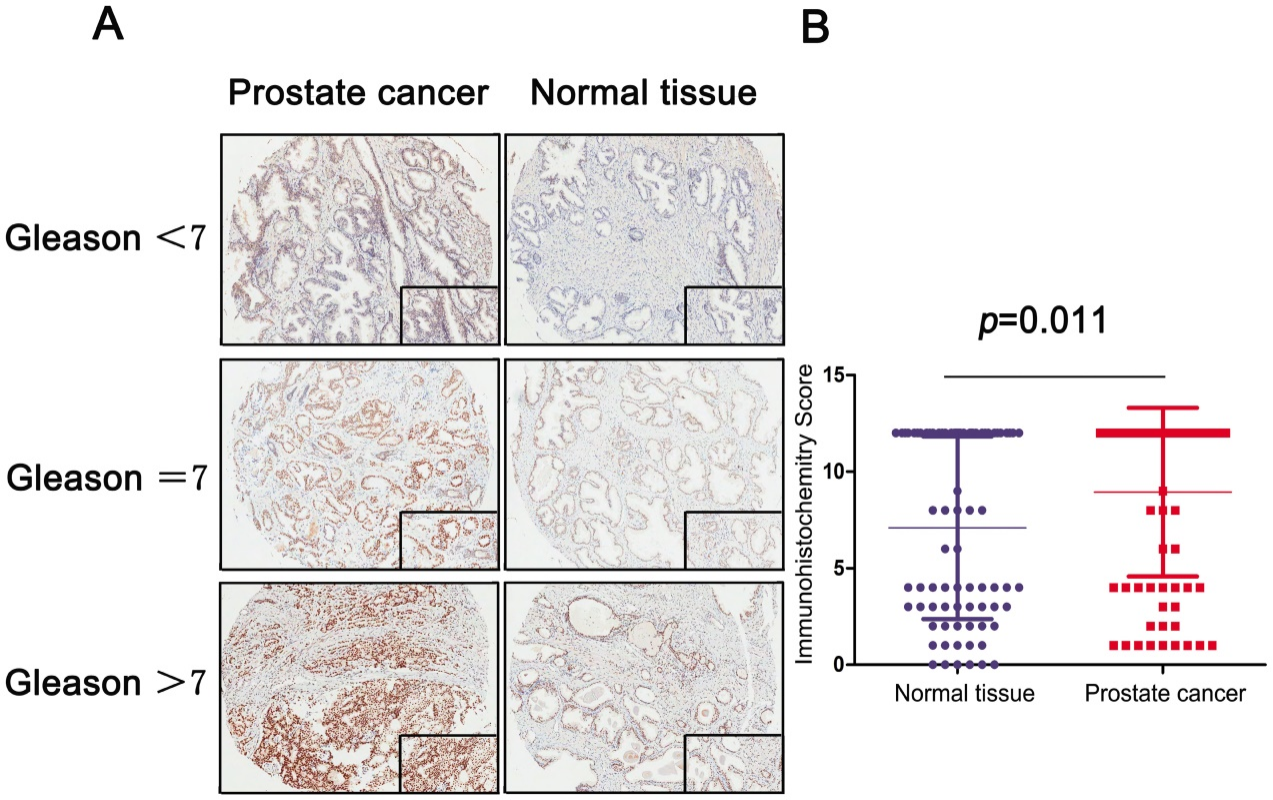
A: IHC stain of prostate and normal tissue in different Gleason score subgroups (Gleason<7, Gleason=7 and Gleason>7); B: Scatter plot of IHC score between normal and tumor tissues (*P*=0.011).

**Figure 2:**
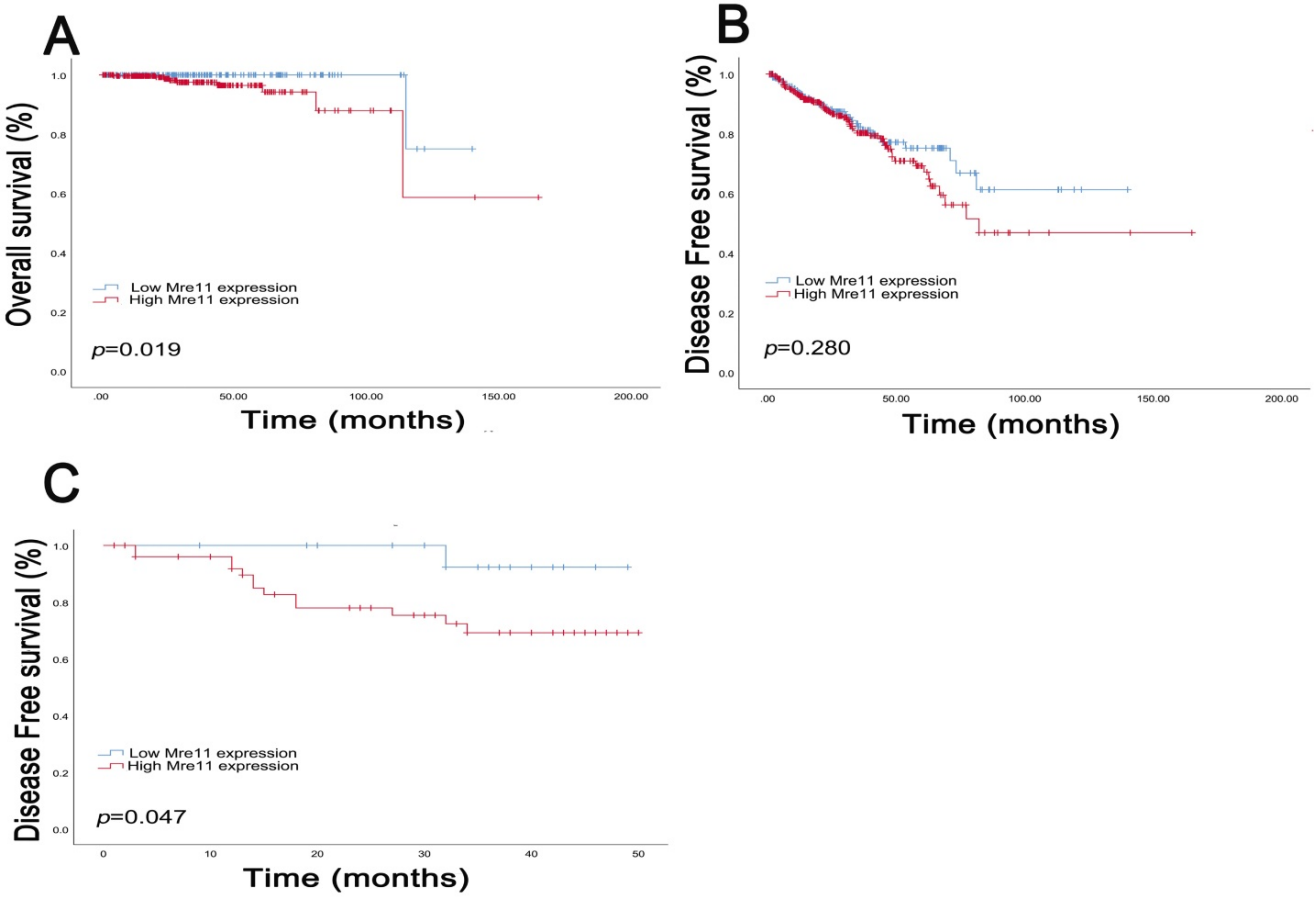
Kaplan-Meier survival analyses on different MRE11 expression groups with OS (A) and DFS (B) in the included 500 PCa patients from TCGA database. Kaplan-Meier survival analyses on different MRE11 expression groups with DFS (C) in the included 78 PCa patients from FUSCC cohort.

**Table 1:** Clinicopathological characteristics in relation to MRE11 expression status in two cohorts

Characteristics	FUSCC cohort (N=78)	MRE11 expression		χ^2^	*P*	TCGA cohort (N=500)	MRE11 expression		χ^2^	*P*
		IHC positive (N=58)	IHC negative (N=20)				IHC positive (N=319)	IHC negative (N=181)		
N (%)										
Age				5.287	**0.021**				0.772	0.380
<65 years	20 (25.6)	11 (19.0)	9 (45.0)			333 (66.6)	208 (65.2)	125 (69.1)		
≥65 years	58 (74.4)	47 (81.0)	11 (55.0)			167 (33.4)	111 (34.8)	56 (30.9)		
Year of diagnosis				1.595	0.450				1.313	0.252
2010	12 (15.4)	9 (15.5)	3 (15.0)			-	-	-		
2011	38 (48.7)	26 (44.8)	12 (60.0)			-	-	-		
2012	28 (35.9)	23 (39.7)	5 (25.0)			-	-	-		
2000-2006	-	-	-			71(14.2)	41(12.9)	30(16.6)		
2007-2013	-	-	-			429(85.8)	278(87.1)	151(83.4)		
Laterality				1.062	0.303				2.291	0.130
Left/Right	17(21.8)	11(19.0)	6(21.8)			65 (13.0)	36 (11.3)	29 (16.0)		
Bilateral	61(78.2)	47(81.0)	14(78.2)			435 (87.0)	283 (88.7)	152 (84.0)		
pT stage				0.258	0.611				2.575	0.109
T1 - T2	35 (44.9)	27 (46.6)	8 (40.0)			195(39.0)	116(36.4)	79(43.6)		
T3 - T4	43 (55.1)	31 (53.4)	12 (60.0)			305(61.0)	203(63.6)	102(56.4)		
pN stage				0.729	0.393				0.043	0.836
N0	61 (78.2)	44 (75.9)	17 (85.0)			348 (69.9)	221 (69.3)	127 (70.2)		
N1	17 (21.8)	14 (24.1)	3 (15.0)			152 (30.4)	98 (30.7)	54 (29.8)		
pM stage				-	-				1.401	0.237
M0	78 (100.0)	58 (100.0)	20 (100.0)			457 (91.4)	288 (90.3)	169 (93.4)		
M1	0	0	0			43 (8.6)	31 (9.7)	12 (6.6)		
AJCC stage †				0.215	0.643				-	-
I - II	65 (83.3)	49 (84.5)	16 (80.0)			-	-	-		
III - IV	13 (16.7)	9 (15.5)	4 (20.0)			-	-	-		
Gleason score				4.312	0.116				9.586	**0.008**
< 7	21 (26.9)	13 (22.4)	8 (40.0)			45 (9.0)	23 (7.2)	22 (12.2)		
= 7	37 (26.9)	27 (46.6)	10 (50.0)			250 (50.0)	150 (47.0)	100 (55.2)		
≥ 8	20 (25.6)	18 (31.0)	2 (10.0)			205 (14.0)	146 (45.8)	59 (32.6)		

† The AJCC staging system is a classification system developed by the American Joint Committee on Cancer for describing the extent of disease progression in cancer patients. It utilizes in part the TNM scoring system: Tumor size, Lymph Nodes affected, Metastases.

**Table 2:** Multivariate Cox logistic regression analysis of DFS in FUSCC and TCGA cohorts (DFS: disease-free survival; FUSCC: Fudan University Shanghai Cancer Center; TCGA: the Cancer Genome Atlas)

Covariates	FUSCC				TCGA		
	HR	95% CI	*P* value		HR	95% CI	*P* value
Age (ref. <65 years)	1.756	0.433-7.125	0.431		1.005	0.972-1.038	0.780
pT stage (ref. T1 - T2)	18.506	2.706-126.551	**0.003**		2.026	1.101-3.728	**0.023**
pN stage (ref. N0)	0.256	0.034-1.933	0.186		0.929	0.592-1.458	0.929
pM stage (ref. M0)	-	-	-		0.475	0.149-1.514	0.208
AJCC stage (ref. I - II)	0.724	0.137-4.124	0.724		-	-	-
Gleason score (ref. <7)	2.518	0.797-7.959	0.116		3.202	3.202-2.013	** <0.001**
MRE11 expression (ref. negative)	8.588	1.015-72.667	**0.048**		1.071	0.687-1.669	0.763

**Table 3:** Univariate and multivariate Cox logistic regression analysis of OS in TCGA cohort (OS: overall survival; TCGA: the Cancer Genome Atlas)

Covariates	Univariate analysis				Multivariate analysis		
	HR	95% CI	*P* value		HR	95% CI	*P* value
Age (ref. <65 years)	1.053	0.955-1.160	0.300		1.064	0.952-1.188	0.276
pT stage (ref. T1 - T2)	3.228	0.598-17.426	0.173		0.739	0.077-7.078	0.793
pN stage (ref. N0)	2.408	0.629-9.226	0.200		1.775	0.365-8.636	0.477
pM stage (ref. M0)	1.958	0.241-15.934	0.530		-	-	0.989
Gleason score (ref. <7)	6.139	1.341-28.100	**0.019**		5.333	0.574-49.553	0.141
MRE11 expression (ref. negative)	8.318	1.031-67.131	**0.047**		9.933	1.042-94.665	**0.046**

## Abbreviations

PCa: Prostate cancer
MRE11: meiotic recombination 11
DSB: DNA double-strand break
IHC: immunohistochemical
TCGA: the Cancer Genome Atlas
FUSCC: Fudan University Shanghai Cancer Center
DFS: disease-free survival
OS: overall survival
HR: hazard ratio
CI: confidence interval
AJCC: American Joint Committee on Cancer
